# Using behavioural science to improve antibiotic stewardship in Canadian long-term care homes: Protocol for a multi-center cluster randomized quality improvement study

**DOI:** 10.14745/ccdr.v51i01a06

**Published:** 2025-01-02

**Authors:** Tyler Good, Jorida Cila, Rhiannon Mosher, Klajdi Puka, Shaghig Reynolds, Barbara Catt, Aboubakar Mounchili, Denise Gravel-Tropper, Patrick Quail, Allison McGeer, Andrea Moser, Madeleine Ashcroft, Peter Daley, Katrina Piggott, Jerome Leis, Mark Morrissey

**Affiliations:** 1 Data, Surveillance and Foresight Branch, Public Health Agency of Canada, Ottawa, ON; 2 Impact & Innovation Unit, Privy Council Office, Ottawa, ON; 3 Infectious Diseases and Vaccination Programs Branch, Public Health Agency of Canada, Ottawa, ON; 4 Department of Family Medicine, Cumming School of Medicine, University of Calgary, Calgary, AB; 5 Department of Laboratory Medicine & Pathobiology, University of Toronto, Toronto, ON; 6 Department of Family and Community Medicine, University of Toronto, Toronto, ON; 7 City of Toronto Senior Services and Long-Term Care Division, Toronto, ON; 8 Trillium Health Partners, Toronto, ON; 9 Newfoundland and Labrador Health Services, Memorial University of Newfoundland, St. John’s, NL; 10 Memorial University of Newfoundland, St. John’s, NL; 11 Sunnybrook Health Sciences Centre, Toronto, ON; 12 Department of Medicine and Centre for Quality Improvement and Patient Safety, Temerty Faculty of Medicine, University of Toronto, Toronto, ON

**Keywords:** antimicrobial resistance, antimicrobial stewardship, education, essential care provider, long-term-care home, audit and feedback, urinary tract infection, urine culture order

## Abstract

**Background:**

Antimicrobial resistance (AMR) is associated with significant human and financial costs, particularly among vulnerable populations like older adults living in long-term care homes (LTCHs). Urinary tract infection (UTI) is the leading indication for antibiotic use in this population, with some estimates suggesting that up to 70% of these prescriptions may be avoidable.

**Objective:**

The purpose of this study is to develop and test novel behavioural science-informed antimicrobial stewardship (AMS) quality improvement strategies in Canadian LTCHs, which aim to decrease unnecessary testing and treatment for residents who lack the minimum clinical signs and symptoms of UTI.

**Intervention:**

The quality improvement strategy is a two-pronged approach that includes 1) targeted education for essential care providers (family and friends of LTCH residents) about UTI and benefits of AMS, which strives to outline a positive role for this group in UTI management, and 2) monthly feedback to LTCH staff on their facility’s urine culture ordering rates.

**Outcomes:**

The protocol was piloted in a single LTCH; a process evaluation of the pilot implementation served to refine the research protocol, which is being implemented in eight LTCHs across Canada using an eight-month stepped wedge randomized cluster design.

**Conclusion:**

This protocol represents a behavioural science-informed intervention to improve AMS across LTCHs. If successful, this model of care could be scalable across Canadian LTCHs, offering an inclusive approach that aims to empower clinicians, non-regulated healthcare staff, residents and their family and friends to improve health outcomes as antibiotic stewards.

## Introduction

The World Health Organization has identified antimicrobial resistance (AMR) as one of the top ten threats to global public health ((1)), with serious human and financial costs ((2)). Some Canadian estimates indicate that up to 50% of antibiotic prescriptions in outpatient settings ((3)), and nearly 25% in hospital settings ((4)), are avoidable. Residents of long-term care homes (LTCHs) are increasingly frail and particularly vulnerable to high rates of antibiotic use and antimicrobial-resistant infections ((5,6)), risk of adverse outcomes linked to avoidable antibiotic use ((7)) and relatively less developed antimicrobial stewardship (AMS) programs compared to other sectors ((8)). The leading indication for antibiotic use in LTCHs is urinary tract infections (UTI) ((9)), as it makes up over half of antibiotics prescribed in this sector ((10)), with up to 70.5% of these prescriptions considered clinically unnecessary ((9)). At the core of this challenge is the occurrence of asymptomatic bacteriuria, which is remarkably prevalent, being present in up to 50% of LTCH residents ((5,11)). Asymptomatic bacteriuria is the expected presence of bacteria in an appropriately collected urine specimen, in absence of clinical symptoms of UTI. Positive urine cultures that identify asymptomatic bacteriuria are frequently attributed to UTI for many non-specific presentations, which underscores the importance of limiting urine culture collection to situations where minimum clinical symptoms are present. An upstream focus on the judicious use of urine cultures is known to result in significant reductions in antibiotic use of asymptomatic bacteriuria ((12,13)) and may significantly improve AMS in LTCHs.

Evidence suggests that AMS interventions in LTCH can reduce antibiotic prescribing, especially for the treatment of UTI ((14–16)), including a recent meta-analysis showing a 14% overall reduction in antimicrobial use ((8)). Upstream interventions targeting urine culture, known as diagnostic stewardship interventions, may be most effective at reducing unnecessary antibiotic prescriptions for UTI ((10,12,13,17–20)). Importantly, a recent systematic review found AMS interventions did not increase risk of hospital admission or death, indicating that these programs did not lead to under-treatment of infection ((21)).

Behavioural science offers a useful lens for addressing antimicrobial resistance ((22)). Behavioural science frameworks have been used to understand the drivers and barriers affecting stewardship behaviours ((23)), as foundation for AMS interventions ((24–28)). In the current work, findings from an initial literature review ((29) were synthesized with stakeholder interview results into a series of mapping exercises that narrowed from a systems, to behaviour, to cognitive map. In this way, we formalized our understanding of how prescribing decisions are influenced by the context of the individual resident, their caregivers, the clinical environment, the healthcare system and the surrounding culture. We then used a barrier prioritization exercise with a working group of experts to identify barriers for our quality improvement (QI) strategies to address. This resulted in development of a two-pronged QI strategy for reducing diagnostic testing and antibiotic treatment of UTI when not clinically indicated. The first strategy consists of targeted education for essential care providers (ECPs; someone who provides important care for a resident and who is not on the medical team, e.g., family member or friend) to address ECP expectations for testing and treatment of UTI when not warranted. The second QI strategy consists of facility-level, monthly feedback about urine culture usage and reminders of guidelines, which will be given to LTCH staff to address the barrier of perceived risk of negative outcomes when choosing non-testing/treatment. Both QI strategies do not require explicit changes to work processes of LTCH staff, an important and advantageous consideration at a time when the Canadian healthcare sector faces human resource challenges.

The effectiveness of the QI strategies will be evaluated by assessing expected reductions in urine culture orders and antibiotic prescriptions for UTI. Whenever possible, we will also examine the proportion of urine cultures aligned with guidelines before and after intervention. A mixed-methods approach will evaluate the success of the study, with qualitative data helping contextualize quantitative findings.

The purpose of this study is to test novel behavioural AMS interventions in support of optimizing testing and treatment of UTI in LTCHs. The primary quantitative research questions are as follows: 1) What is the baseline usage of urine cultures in participating LTCHs?; and 2) Does implementation of the proposed QI strategies reduce the rates of a) urine cultures, b) antibiotic prescriptions for UTI and c) overall antibiotic prescriptions? Exploratory research questions will examine the baseline proportion of urine cultures aligned with guidelines and what risk factors are associated with collection of urine cultures when not aligned with guidelines. Qualitative data will also be collected to nuance quantitative findings.

## Methods

### Study overview

The study will be conducted in two stages. In the Pilot Stage, the protocol was implemented in a single LTCH for process evaluation (see **Appendix A: Protocol refinements**). The Trial Stage involves implementation in eight other LTCHs across Canada, with the main objective being an outcomes evaluation. The Trial Stage is designed as a stepped-wedge cluster randomized quality improvement study (**Table 1**) using a mixed-method approach. Quantitative data will evaluate the effectiveness of the protocol at reducing both testing and treatment for UTI, and qualitative data will contextualize the findings. Long-term care homes will be randomized to different starting times for the crossover from the control to intervention phases, with staff and residents blinded to their allocation sequence. Here, we present the final protocol, including changes informed by Pilot Stage findings. For a complete list of refinements made to the protocol following the Pilot Stage, refer to Appendix A: Protocol refinements.

**Table 1:** Overview of the stepped-wedge design

LTCH	Cluster	Month 1	Month 2	Month 3	Month 4	Month 5	Month 6	Month 7	Month 8
a	1	C	C	T	I	I	I	I	I
b	1	C	C	T	I	I	I	I	I
c	2	C	C	C	T	I	I	I	I
d	2	C	C	C	T	I	I	I	I
e	2	C	C	C	T	I	I	I	I
f	3	C	C	C	C	T	I	I	I
g	4	C	C	C	C	C	T	I	I
h	4	C	C	C	C	C	T	I	I

Abbreviations: C, Control Phase (usual care is given); I, Intervention Phase (implementation of intervention); LTCH, long-term care home; T, Transition Phase (initiation of intervention)

### Sample characteristics

A purposive sampling strategy was used to recruit large (approximately 200 residents) LTCHs across Canada. To be eligible, LTCHs had to provide long-term (permanent placement) residential care with 24-hour monitoring and medical assistance. Sample size calculations using Hemming and Taljaard’s approach ((30)) indicated that eight LTCHs across four clusters, observed for a total of eight months, would be sufficient to detect a clinically meaningful 20% reduction in rate of urine culture ordering (6.5 to 5.2 urine cultures per 1,000 resident days) at greater than 80% power and 5% significance level. The effect size is in line with previous studies that observed greater than 25% reduction in urine culture ordering ((31,32)) and is more conservative than 25% reduction used in sample size calculations for a similarly designed trial ((33)).

Two limitations of this trial are its smaller sample size and the purposive sampling technique, which will not provide a representative sample of LTCHs across Canada. However, this is the first pilot study of a novel intervention, so the smaller more homogenous sample will provide initial evidence on the effectiveness of the strategies, which will allow for improvement of the processes and materials.

### Pilot Stage

The Pilot Stage took place in a single LTCH from May to August 2023, starting with retrospective data collection (for the period February 2022 to January 2023), continuing with a transition phase where the QI strategies were brought online and concluding with a one-month intervention phase. The main output from the Pilot Stage was a process evaluation to gather exploratory and evaluative insights to validate study materials, check assumptions, identify gaps in the interventions and evaluate in-field processes. A series of semi-structured interviews with LTCH staff (n=3), a focus group with ECPs (n=2), voluntary online surveys (n=10) and direct observation of the materials deployed in the home informed the process evaluation. In addition to these targeted sources of information input from the frontline workers of the Local Implementation Team was valuable to ground and validate our analytical interpretations in a deeper understanding of the local context of the home ((34–36)).

Fewer ECPs participated in the focus group than anticipated and this was likely at least partially due to the necessary timing of the pilot during the summer months, especially as the regular touchpoint of the resident family and friends council was on hiatus. However, even these few responses provided a valuable level of nuance regarding ECPs’ perceptions of the educational materials and of UTI treatment best practices that helped to identify areas for consideration and improvement for the stepped-wedge trial.

### Retrospective data collection

Facility and demographic data will be collected along with proposed outcomes metrics for a retrospective one-year period to provide historical insight, contextualize these data with home demographics and serve as true baseline for comparison with intervention phase data. Participating LTCHs will be provided detailed data dictionaries and template data entry forms to ensure consistency.

### Control Phase

The Control Phase will last between two and five months, depending on cluster number (Table 1). During the Control Phase, usual care will be given to the LTCH residents, and minimum and additional data elements (such as the number of urine cultures ordered and catheter use) will be collected on a monthly basis, as necessary, to answer both the primary and exploratory research questions. The complete list of variables is provided in **Appendix B: Outcome metrics**.

### Transition Phase

During the one-month Transition Phase, the research team will liaise with each LTCH to coordinate education and delivery of the interventions. The goals of the Transition Phase are to 1) provide level-setting foundational AMS knowledge and practices to help standardize the intervention across participating LTCHs and 2) coordinate the implementation of the intervention. To further ensure alignment, the research team will offer to connect the medical leadership of each LTCH with a physician member of the study working group for an optional peer-to-peer conversation about the guidelines and their experience with implementation within their practice.

During this phase the research team will deliver brief education sessions for LTCH staff (nurses, physicians, non-regulated healthcare staff, pharmacists) about the overprescribing of antibiotics, a reminder of when it is and is not appropriate to test for and treat UTI in older adults ((37,38)) and practices that can contribute to this problem. These sessions will be delivered in-person or by webinar at the discretion of the LTCHs. A recording will be made available for new staff and those unable to attend the synchronous sessions.

### Intervention Phase

The Intervention Phase will consist of two primary strategies: 1) ECP education and 2) monthly feedback letters to LTCH staff about facility urine culture ordering.

**Essential care providers education:** Although educational components are common in AMS interventions within LTCHs ((14,18,38–46)), they typically target physicians and/or nurses. Fewer studies have provided education for ECPs ((11)), despite ECPs’ influence on testing and treatment decisions ((16,47–49)). We designed these educational resources to increase understanding of AMS and the harms of unnecessary antimicrobial use among ECPs, and to outline a positive advocacy role for ECPs in UTI management.

Drawing on lessons learned from the Pilot Stage, taking a multi-modal approach to ECP education will help reach a broader audience among this diverse target population. Brief education sessions will be delivered in-person by a LTCH staff trained by the research team and asynchronously by leveraging digital communications and in-home communications (e.g., UTI best practice posters in common areas). In-person and live virtual sessions will be offered monthly, with exact frequency to reflect each LTCH’s unique needs, and delivered within regularly occurring events (e.g., monthly LTCH town halls). Educational materials will also be distributed to ECPs through videos, posters, newsletters and physical handouts made available at the LTCH. We hypothesize that this intervention will reduce urine culture ordering and antibiotic prescribing by increasing ECPs’ knowledge about AMS and, therefore, decreasing caregiver expectations for these tests and treatments when not clinically indicated.

**Feedback letter:** This strategy consists of monthly feedback given to LTCH staff (i.e., nurses, non-regulated healthcare staff, pharmacists, physicians) that shows the rate of urine cultures ordered by their facility in the past month relative to their previous data (retrospective, Control, Transition and previous Intervention Phase when relevant). Audit and feedback on antibiotic prescription use have been embraced for use with physicians ((50)). Feedback to nurses and non-regulated healthcare staff, however, has not been used as an intervention strategy in LTCHs, yet these professionals play particularly important roles in LTCHs. They collaborate with physicians in making these decisions typically by assessing the resident and communicating their observations to the physician and, in some cases, collecting a urine sample before the physician has assessed the resident ((49,51-53)). Comparing recent with past performance acts as a self-comparison, which can motivate recipients by establishing personal norms ((54)) and has been effective in other contexts ((55)). Feedback will also indicate the proportion of urine cultures aligned with best practice guidelines (for LTCHs able to collect signs and symptoms data), which is a more specific measure of stewardship than overall ordering rate alone ((56)). We hypothesize that the feedback strategy will increase institutional awareness and reduce perceived risk of negative outcomes of urine culture avoidance, ultimately leading to a decrease in urine cultures and antibiotic prescriptions.

Feedback will be provided to all LTCH staff (nurses, non-regulated healthcare staff, pharmacists, physicians) starting after the first month of the Intervention Phase, for a total of two to five cycles of feedback depending on the cluster number. The LTCH implementation team will work with the research team to identify appropriate medium(s) for this feedback (e.g., central communications location on the floor, email, bulletin boards, regular staff meetings). The feedback letter will also include reminders regarding urine culture ordering decision guidelines ((37,38)) and links to additional resources.

### Study measures

De-identified quantitative data will be collected monthly during the Control, Transition and Intervention Phases. Minimum data elements needed to answer the primary research questions include number of urine cultures ordered, antibiotic prescriptions for UTI, total antibiotic prescriptions and total days of residence. Additional data elements are necessary to answer the exploratory research questions and include signs and symptoms prompting urine culture orders, resident demographic characteristics, chronic conditions and functional status.

To contextualize the quantitative findings with the perspectives of the end users (LTCH staff and ECPs), we will additionally 1) conduct semi-structured interviews with 3–6 staff members from each LTCH after the Intervention Phase; 2) hold 2–3 focus groups with 4–6 ECPs each, within two months of the end of the intervention phase; and 3) collect qualitative data on perceptions and experience with the study through voluntary online questionnaires available to all LTCH staff and ECPs throughout the study. As with the Pilot Stage, qualitative data collection and validation will be supported by Local Implementation Teams.

### Data analysis

A series of descriptive (continuous variables) and frequency analyses (categorical variables) will be conducted to get a global sense of the sample responses.

Analysis will be done at the level of the LTCH as an intention-to-treat analysis. To evaluate research question 1, rate of urine culture (per 1,000 resident days) will be calculated for the retrospective data period. To evaluate research question 2a, a generalized linear mixed-effects model will be used to assess whether or not the intervention has an effect on the rate of urine culture (per 1,000 resident days). The model will include categorical, fixed effects for phase (control/intervention) and for each month to account for secular trends, as well as a random effects for LTCH. Data from the Transition Phase will be excluded from these analyses as we do not consider these data to be clearly Control or Intervention Phase data. To evaluate research questions 2b and 2c, a similar model will be used with the outcome measures rate of antibiotic prescriptions for UTI and total antibiotic prescriptions per 1,000 resident days. Exploratory analyses will use a similar model to evaluate the potential effect of the intervention on rate of urine cultures not aligned with guidelines. Alignment with guidelines will be estimated by comparing the signs and symptoms prompting each urine culture to meet the modified Loeb minimum criteria for UTI for a catheterized or non-catheterized resident ((37,38)).

Thematic analysis will be guided by ethnographic methods and Normalization Process Theory. Taking an iterative approach that draws on Grounded Theory ((35)), open-ended codes will be applied alongside selected evaluative codes developed from a Normalization Process Theory perspective ((57,58)). Ethnographic data reduction techniques will be applied to surface focused insights to support the overarching research questions for the study ((34,36,59)).

### Refinements to intervention based on Pilot Stage

A series of key observations drawn from the suite of qualitative methods employed were noted during the Pilot Stage: most staff who participated in interviews and ECPs who participated in the questionnaire and/or focus group viewed UTI guidelines as only relevant to residents without dementia, despite the guidelines’ validation in LTCHs with residents with and without dementia ((37,38)). To address this challenge, two adjustments were made to the protocol: 1) the addition of an optional peer-to-peer conversation between the medical leadership of the LTCH and a physician member of the study’s expert working group and 2) the revision of educational materials to highlight validation of guidelines in LTCHs and share experience in management of residents with dementia, including those that are non-communicative. For a full accounting of revisions in response to preliminary findings, see Appendix A.

Findings from the questionnaire, focus group and observations by staff interviewees and the Local Implementation Team indicated that the educational component of the intervention was generally appreciated by the ECPs, seeing it as relevant to their role as caregiver. However, it was observed across our qualitative data that ECPs constituted a heterogenous group and scheduling was, at times, challenging. Therefore, we increased our flexibility to offer multimodal delivery of educational materials (i.e., poster, handout, in-person session, video).

Regarding the feedback letter, members of the Local Implementation Team and all interviewed staff (n=3) expressed uncertainty about the intended use and action. Some concerns regarding the peer-comparison were also raised, highlighting the challenge of inter-home comparisons. To address these challenges, the following changes to the protocol were made: 1) inclusion of an estimate of proportion of urine cultures aligned with guidelines to provide a more actionable metric, 2) shift from peer-comparison to self-comparison to emphasize continual self-improvement and 3) highlighting links to additional supports and resources.

## Conclusion

This study will rigorously evaluate the impact of a behavioural science-informed intervention to improve AMS across LTCHs. If successful, this model of care could be scalable across Canadian LTCHs, offering an inclusive approach that aims to empower clinicians, non-regulated healthcare staff, residents and their family and friends to improve health outcomes as antibiotic stewards.

## Appendices

### Appendix A: Protocol refinements

The following table summarizes changes made to the protocol following the Pilot Stage. The protocol described in the paper reflects these changes.

**Table:**

Change	Change made	Description
1	Addition of an optional peer-to-peer conversation between physician member of the study expert working group and medical leadership at each home at the beginning of the Transition Phase	A goal of our Transition Phase is level-setting (getting everyone on the same page) about foundational AMS knowledge and practices. To facilitate this, we meet with the implementation team at each LTCH and review current practices and alignment with guidelines. To strengthen this, the protocol now includes an optional peer-to-peer conversation between a physician member of the working group and the medical leadership at each LTCH (i.e., Medical Director, Physician Chief). The intention is for these conversations to cover the evidence supporting the guidelines and the experiences the working group member had in implementing them in their practice.
2	Explicit inclusion of clinical pharmacists and personal support workers	The protocol now calls for clinical pharmacists employed by the home and non-regulated healthcare staff (sometimes referred to as personal support workers or nursing assistants) to attend the introductory education session provided during the Transition Phase, along with nursing staff and physicians. Previously these groups were not explicitly named in our protocol, despite being likely to interact with study materials present in staff areas (e.g., a monthly feedback report at the nursing station). This change appropriately includes them as important members of the clinical team that a portion of the responsibility for AMS.
3	Increased flexibility in delivery of staff introductory education session	The duration of the introductory education session was reduced to 5−10 minutes and the protocol calls for it to be presented in-person, by webinar or via recorded video at the discretion of the LTCH. This increased flexibility is intended to allow for the adaption to the unique circumstances and procedures of staff training at each LTCH. The session is now provided to nurses, physicians, clinical pharmacists and non-regulated healthcare staff (personal support workers, nursing assistants, etc.), as per change 2.
4	Narrowing of prospective data collection	The protocol now includes collection of signs and symptoms prompting all urine culture orders during the Control, Transition and Intervention Phases. This allows an estimation of the proportion of cultures that are aligned with guidelines. A measure of alignment with guidelines will allow for a more precise measure of AMS compared to rate of urine culture alone. Alignment with guidelines will be used as an exploratory evaluation of the success of the trial (pre-post comparison) as well as a component of the feedback report. To balance the additional workload to LTCHs collecting this data, the protocol also limits the previously required monthly facility-level demographic data from all residents of the LTCH to only those who received a urine culture. The previous monthly facility-level demographics provided little additional value to the cross-sectional demographic data collected with the retrospective data. If some LTCHs are unable to provide signs and symptoms data, we will 1) report this finding, which will highlight an important knowledge gap, 2) remain adequately powered to evaluate the study using rate of urine culture order and 3) antibiotic prescription for UTI as previously planned.
5	Updates to feedback letter content	The protocol now calls for the feedback letter to provide a self-comparison of LTCH urine culture order rate over time, as well as an estimate of the proportion of urine culture orders that were aligned with guidelines. Previously, the protocol called for a peer-comparison of urine culture order rate between the LTCHs included in the Trial Stage, as well as comparison with historical data. The change avoids limitations of inter-home comparisons and allows for a focus on self-comparison in the spirit of continual improvement.
6	Increased opportunity for qualitative engagement with staff	The protocol calls for a minimum of three, but opportunity for six semi-structured interviews at each LTCH. This is a change from the previous protocol which required three interviews, with no opportunity for more. The protocol also includes a voluntary online questionnaire available to LTCH staff. This mirrors the questionnaire provided to ECPs, asking about LTCH staff’s experience with our study and suggestions for improvements. Together, these changes provide opportunity to supplement qualitative findings from a previously small number of interviews, only if there is interest and capacity at each LTCH. The online questionnaire provides a mechanism for all LTCH staff to share feedback, if they choose to do so.

Abbreviations: AMS, antimicrobial stewardship; ECP, essential care provider; LTCH, long-term care home; UTI, urinary tract infection

### Appendix B: Outcome metrics

The intervention study will be collecting data on the following key outcomes:

1. Outcomes related to urine culture orders

a. Baseline prevalence of urine cultures for diagnosis of urinary tract infections (UTIs)

b. Effect of intervention on decreasing rate of urine culture orders

c. Effect of intervention on decreasing rate of urine culture orders aligned with guidelines

2. Outcomes related to antibiotic use

a. Baseline usage of antibiotics

b. Effect of intervention on reducing incidence of antibiotic prescriptions written for suspected UTIs

c. Effect of intervention on reducing duration of written urinary antibiotic prescriptions

d. Effect of intervention on reducing incidence of total antibiotic prescriptions

e. Effect of intervention on duration of total antibiotic prescriptions

3. Outcomes related to essential care provider (ECP) education

a. Long-term care home (LTCH) staff perceptions on whether ECP education reduced pressure from ECPs to collect urine cultures for testing when not clinically indicated

b. LTCH staff perceptions on whether ECP education reduced pressure from ECPs for antibiotic treatment of UTIs when not clinically indicated

4. Outcomes related to feedback letter

a. LTCH staff perceptions on whether the feedback letter reduced their perceived risk of negative outcomes when not ordering diagnostic testing or treatment for UTIs when not clinically indicated

In addition to the above, we will also be collecting data on additional outcomes to test some exploratory research questions (e.g., result of urine culture; antibiotic dosage, duration and route of administration; catheter use; ethnicity; sex; age; chronic conditions).
